# Recanalization status and temporal evolution of early ischemic changes following stroke thrombectomy

**DOI:** 10.1177/23969873231214207

**Published:** 2023-11-22

**Authors:** Pekka Virtanen, Liisa Tomppo, Georgios Georgiopoulos, Nina Brandstack, Erno Peltola, Tatu Kokkonen, Kimmo Lappalainen, Antti Korvenoja, Daniel Strbian

**Affiliations:** 1 Department of Radiology, Helsinki University Hospital and University of Helsinki, Helsinki, Finland; 2 Department of Neurology, Helsinki University Hospital and University of Helsinki, Helsinki, Finland; 3 Department of Clinical Therapeutics, National and Kapodistrian University of Athens, Athens, Greece; 4 School of Biomedical Engineering and Imaging Sciences, King’s College London, London, UK

**Keywords:** Early ischemic signs, computed tomography, thrombectomy, recanalization

## Abstract

**Introduction:**

Present-day computer tomography (CT) scanners have excellent spatial resolution and signal-to-noise ratio and are instrumental detecting early ischemic changes (EIC) in brain. We assessed the temporal changes of EIC based on the recanalization status after thrombectomy.

**Patients and methods:**

The cohort comprises consecutive patients with acute ischemic stroke in anterior circulation treated with thrombectomy in tertiary referral hospital. All baseline and follow-up scans were screened for any ischemic changes and further classified using Alberta Stroke Program Early CT Score (ASPECTS). Generalized linear mixed models were used to analyze the impact of recanalization status using modified Thrombolysis in Cerebral Infarction (mTICI) on temporal evolution of ischemic changes.

**Results:**

We included 614 patients with ICA, M1, or M2 occlusions. Median ASPECTS score was 9 (IQR 7–10) at baseline and 7 (5-8) at approximately 24 h. mTICI 3 was achieved in 207 (33.8%), 2B 241 (39.3%), 2A in 77 (12.6%), and 0–1 in 88 (14.3%) patients. Compared to patients with mTICI 3, those with mTICI 0–1 and 2A had less favorable temporal changes of ASPECTS (*p* < 0.001). Effect of recanalization was noted in the cortical regions of ICA/M1 patients, but not in their deep structures or patients with M2 occlusions. All ischemic changes detected at baseline were also present at all follow-up images, regardless of the recanalization status.

**Conclusions:**

Temporal evolution of the ischemic changes and ASPECTS are related to the success of the recanalization therapy in cortical regions of ICA/M1 patients, but not in their deep brain structures or M2 patients. In none of the patients did EIC revert in any brain region after successful recanalization.

## Graphical abstract

**Graphical abstract:**
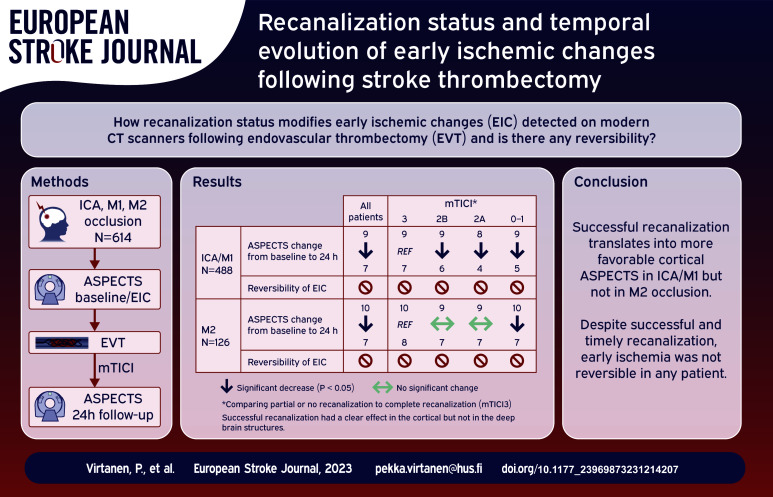

## Introduction

There is a plethora of information about evolution of EIC into the final infarct size in experimental and clinical ischemic stroke.^1–3^ Major factors determining the tissue fate include severity of reduction of cerebral blood flow following the vessel occlusion, duration of the occlusion (hence, successful delivery of the recanalization therapy or spontaneous recanalization), but also status of the collateral flow.^4^

EIC observed on non-contrast computed tomography (NCCT) scans were conventionally considered to be irreversible.^5^ However, it remains unclear whether such changes that are visible on modern CT scanners (as compared to the previous generations of the scanners) can be reversible as a result of successful recanalization. Such debate was driven by substantial innovation of CT imaging techniques in the recent years and by few reports of partial reversal of diffusion-weighted imaging (DWI) changes following successful recanalization.^6^ Indeed, current CT scanners are highly sensitive and have good signal to noise ratio thanks to advancements in detector technologies and image reconstruction algorithms.^7,8^

We aimed to study temporal changes of EIC using Alberta Stroke Program Early CT Score (ASPECTS) as related to the recanalization status following endovascular thrombectomy (EVT). We hypothesized that better recanalization translates into higher ASPECTS scores at 24 h compared to patients with suboptimal recanalization. Our secondary aim was to address reversibility of EIC after successful recanalization.

## Patients and methods

### Patient cohort

Our study cohort includes all consecutive patients who underwent EVT at the Helsinki University Hospital, Finland. Because the collection of radiologic data was tedious, we restricted the study period (from January 2018 to December 2020). In the present study, we included patients with occlusion in the anterior circulation (ICA, M1, M2). The stroke symptoms were assessed by stroke neurologist using the National Institutes of Health Stroke Scale (NIHSS) at baseline (prior to EVT) and at 24 h post EVT. Relevant clinical parameters and demographic details including age at onset, sex, and time when last seen well (LSW) were obtained from electronic medical charts. Further, time from arrival to recanalization was recorded. In our institute patients come directly to CT room upon arrival. Hence, time of arrival is only a few minutes prior to baseline imaging, and we used it as a surrogate for the imaging time. Minimum data for all patients were prospectively included into the study database, however, some (mostly radiological) parameters were obtained retrospectively by the study radiologists.

Our study was approved by the Institutional Review Board. Ethical review or patient consent was not required for the analysis of the data collected as a part of routine clinical care.

### Imaging and scanners

Our stroke imaging protocol includes NCCT, computer tomography angiography (CTA) and computer tomography perfusion (CTP). All scans were acquired by modern multidetector CT scanners (Siemens Definition Edge, 128 slice detector, and Siemens Somatom Force, 2x198 slice detector: Siemens, Erlangen, Germany; GE Revolution EVO, 128 slice detector, GE Healthcare, Milwaukee, WI). Imaging parameters for NCCT: Modulation for GE: 120 kV, Noise Index 9. Modulation for Siemens: 120 kV, ref mAs 410. Reconstruction thickness 0,625 mm. About 0.6 mm axial images and 3 mm axial, sagittal and coronal plane images were reconstructed. Imaging parameters for CTA: Modulation for GE: 120 kV, Noise Index 14. Modulation for Siemens: 120 kV, ref mAs 200/11. Reconstruction thickness 0.625 mm. About 0.6/0.3 mm axial images and 22/3 mm maximum intensity projection (MIP) in axial, coronal and sagittal planes were reconstructed. Imaging parameters for CTP: GE: 80 kV, 150 mA, imaging interval 1.3 s, imaging time 49 s. Siemens: 70 kV, 135 mAs, imaging interval 1.5 s, imaging time 45 s.

Digital subtraction angiography (DSA) was done using Siemens mono- or biplane system (Siemens Icono Biplane, Siemens Artis Q). EVT was performed by stroke interventional radiologists. Modern third generation stent-retrievals were used along with procedure techniques such as Solumbra, ADAPT, or their redefined versions. Thrombus location was evaluated from baseline CTA and re-evaluated at the beginning of EVT from DSA. Recanalization status from post-EVT DSA images was evaluated with reference being occlusion location at the beginning of EVT and scored by the study radiologists according to the modified Thrombolysis in Cerebral Infarction (mTICI).^9^

Post-treatment CT or MRI was obtained approximately 24 h after EVT and whenever clinical deterioration occurred and/or intracranial hemorrhage (ICH) was suspected. Follow up imaging was done by CT in 587 (96%) patients and by MRI in 27 (4%) patients (who underwent CT follow-up at later time points). Post-EVT scans were acquired on Siemens CT scanners (Somatom EDGE or Force) or 1.5 T Siemens MRI scanner (Magnetom Avanto Fit).

### Early ischemic signs and ASPECTS

Baseline NCCT was used to evaluate presence and location of EIC. Briefly, cortical and deep gray matter hypoattenuation was considered as ischemic. These changes were further classified according to the ASPECTS scoring system,^10^ where 10 standardized brain regions are evaluated and one point is reduced for each region with visible ischemia.^11^

Post-EVT ASPECTS was scored from follow-up NCCT or MRI at approximately 24 h after EVT, without knowledge of the baseline ASPECTS. Thereafter, all post-EVT CT scans (including those from later stages of hospitalization) were carefully evaluated for presence of ischemia and compared to the baseline scans. On 24 h MRI, areas with diffusion restriction in DWI imaging ware considered ischemic. If follow-up imaging was done, we assessed if it was due to clinical deterioration and if the patient had an ICH or a new ischemic event, which could impact the interpretation of the index ischemia’s evolution.

All scans were reviewed by experienced neuroradiologist (PV) with over 10 years of expertise in neuroradiology. To obtain inter- and intra-rater variability, a subset of CT scans from 30 randomly selected patients was assessed by independent neuroradiologist (NB) with over 10 years of expertise in neuroradiology, and again by the study neuroradiologist (PV) – both in a blinded fashion.

Radiological data were analyzed on a PACS (picture archiving and communication system) workstation (AGFA IMPAX; Agfa HealthCare, Belgium and Syngo.plaza and Syngo.share, Siemens, Germany).

### Statistical analyses

Data are shown as median and interquartile range (IQR) for the continuous variables and as percentages for the binary and categorical ones. We used generalized linear mixed models (GLMM) to assess changes in ASPECTS from baseline to 24 h post-recanalization. In specific, multi-level mixed-effects ordered logistic and logistic regression models with random intercept and an independent variance-covariance matrix were employed to assess ischemic changes in repeated measurements (at baseline and 24 h), both for total ASPECTS and then separately for the subregions of ASPECTS. Finally, to assess differential responses in ASPECTS according to achieved level of recanalization, we tested the interaction between the mTICI grading and index measurement (pre- and post-recanalization). Next, we repeated the analysis of temporal ASPECTS changes separately in the ICA/M1 and M2 subcohorts. Inter- and intra-rater correlation were evaluated using the intraclass correlation coefficient (ICC). We did not perform correction for multiple comparisons as distinct GLMMs were employed per pre-specified subcohort of patients or brain region (independent hypotheses). Data were analyzed using the SPSS Statistics software version 25 (IBM Corp, Armonk, NY, USA). All tests were two-tailed. We deemed statistical significance at *p* < 0.05.

## Results

Altogether, 614 stroke patients underwent EVT. Their median age was 71 [IQR 62–79] years and 45% were females. Baseline NIHSS was median 14 [8–18]. Time from last-seen-well to arrival was median 170 [73–347] minutes. Site of occlusion was ICA or M1 in 488 patients (79.5%) and M2 in 126 (20.5%). Post-EVT mTICI 2B/3 was achieved in 448 (73.1%), 2A in 77 (12.6%), and 0–1 in 88 (14.3%) patients. Median time from arrival to recanalization was 103 [IQR 80–135] minutes in ICA/M1 subcohort and 103,5 [IQR 79–132.5] minutes in M2 subcohort. The 10th percentile was 62 min in both groups.

ASPECTS could not be reliably evaluated in 21 baseline and 31 control CT scans. At baseline, this was mostly due to chronic ischemic changes or poor image quality, whereas it was due to hemorrhage or lack of control imaging due to death at 24 h. The cases with missing scans were excluded from the analysis of temporal changes.

### Temporal changes of ischemia (baseline vs 24 h) after thrombectomy

**Figure 1.**
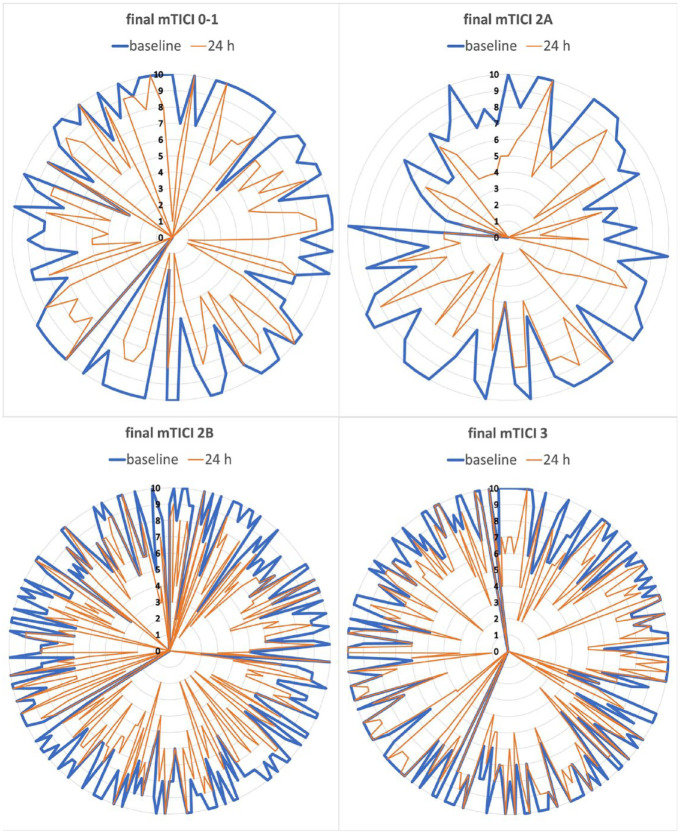
Extent of ischemic signs according to ASPECTS from baseline to 24 h based on recanalization status.

Temporal changes of ASPECTS based on recanalization status are visualized in Figure 1. Presence of ischemic changes at baseline and at 24 h in ASPECTS subregions are shown in Table 1. The ischemic changes at baseline were most frequent in insular ribbon, followed by basal ganglia and the same pattern was seen at 24 h.

**Table 1.** Presence of ischemic changes in subregions of ASPECTS at baseline and 24 h.

Region	Baseline (*n* = 593) (%)	24 h (*n* = 583) (%)
Absence of cortical ischemia	67.8	25.4
Ischemia in 1 cortical region	15.2	22.6
Ischemia in 2 cortical regions	9.6	19.0
Ischemia in 3 cortical regions	4.9	10.3
Ischemia in 4 cortical regions	0.8	8.7
Ischemia in 5 cortical regions	0.5	7.2
Ischemia in 6 cortical regions	1.2	6.7
Insular ribbon	42.7	65.5
Lentiform nucleus	27.3	51.1
Caudate nucleus	24.3	50.6
Internal capsule	1.2	10.1

### Total ASPECTS

In the whole cohort (all occlusion locations and irrespective of recanalization status), ASPECTS significantly decreased between baseline (9 [7–10]) and 24 h (7 [5–8], *p* < 0.001). In the ICA/M1 subcohort, the decrease of ASPECTS was also significant: ASPECTS at baseline 9 [7–10] and ASPECTS at 24 h 7 [4–8] (*p* < 0.001). The same was true for the M2 subcohort: ASPECTS at baseline 10 [8–10] and 24 h 7 [6–8] (*p* < 0.001). There was no patient with more favorable ASPECTS at 24 h as compared to baseline. ASPECTS remained stable in control imaging in 126 (22%) and declined by 1 point in 134 (24%) patients.

Temporal changes of ASPECTS according to the recanalization status are shown in Table 2 and in Figure 1. Compared to patients with post-EVT mTICI 3, those with mTICI 0–1 and 2A had a significant deterioration of ASPECTS (both *p* < 0.001), however, only a trend was observed for mTICI 2B (*p* = 0.067). Very similar results were observed in the ICA/M1 subcohort, where the patients with mTICI 0–1 and 2A had a significant decrease in ASPECTS (both *p* < 0.001), whereas a lesser significance (*p* = 0.034) was observed for mTICI 2B. However, in the M2 subcohort, only the patients with mTICI 0–1 had a significant decline in ASPECTS (*p* = 0.030) compared with mTICI 3.

**Table 2.** Temporal changes of total ASPECTS according to the recanalization status.

	ASPECTS		*p*-Value
	Baseline	24 h	
All patients (ICA, M1, M2)			
Post-EVT mTICI 0–1	9 (8, 10)	6 (4, 8)	<0.001
Post-EVT mTICI 2A	8 (7, 10)	5 (4, 7)	<0.001
Post-EVT mTICI 2B	9 (7, 10)	6 (5, 8)	0.067
Post-EVT mTICI 3	9 (8, 10)	7 (6, 9)	Reference
ICA or M1			
Post-EVT mTICI 0–1 (13%)	9 (8, 10)	5 (2, 8)	<0.001
Post-EVT mTICI 2A (11%)	8 (6, 10)	4 (3, 5)	0.001
Post-EVT mTICI 2B (41%)	9 (7, 10)	6 (5, 8)	0.034
Post-EVT mTICI 3 (35%)	9 (8, 10)	7 (5, 9)	Reference
M2			
Post-EVT mTICI 0–1 (20%)	10 (9, 10)	7 (6, 8)	0.030
Post-EVT mTICI 2A (19%)	9 (8, 10)	7 (5, 7)	0.148
Post-EVT mTICI 2B (32%)	9 (7, 10)	7 (6, 8)	0.668
Post-EVT mTICI 3 (29%)	10 (8, 10)	8 (6, 9)	Reference

Data are presented as median (25th and 75th percentile). The *p*-values come from GLM.

We analyzed the impact of the first-pass effect in those patients who achieved mTICI 2B or 3 within 1 h from arrival. The temporal evolution of ASPECTS in these patients was more favorable than in the rest of the patients (data not shown), but we did not observe any reversibility of EIC occurred.

### Subregions of ASPECTS

Temporal ischemic changes in the individual ASPECTS subregions are outlined in Table 3. Regarding whole cohort, we observed a significant change toward more frequent ischemia at 24 h in all subregions of ASPECTS irrespective of the recanalization status. However, when the recanalization status was included in the analysis, the effect of successful recanalization was seen only in the cortex (Table 3, mTICI 3 as reference). Very similar results were observed in the ICA/M1 subcohort. However, in the M2 subcohort, the recanalization status did not have any effect on the temporal changes of ischemia in any region (Table 3). We have not observed any reversal of ischemia in any subregion of ASPECTS.

**Table 3.** Temporal ischemic changes in subregions of ASPECTS according to the recanalization status.

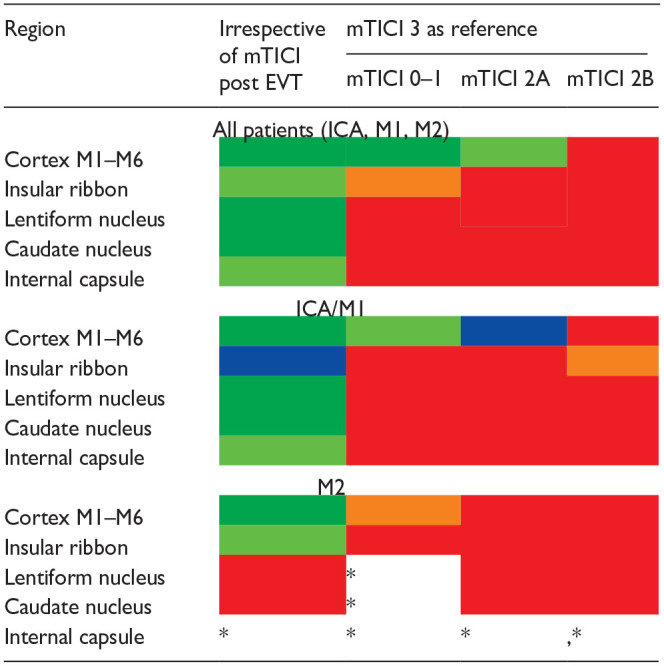

Dark green: *p* < 0.001; light green: *p* < 0.01; blue: *p* < 0.05; orange: p = 0.05–0.10; red: *p* > 0.10.

* Not enough observations.

Footnote: The table shows effect of complete recanalization (mTICI 3) on the temporal evolution of EIC in the ASPECTS subregions as compared to other levels of recanalization categories. Results are shown for all patients and separately for ICA/M1 and M2 patients.

### Other brain regions

In addition to ASPECTS, presence or absence of ischemia was also evaluated in all other brain regions (including ACA and PCA territory). No reversal of ischemia was observed at 24 h as compared to the baseline.

### Intra-rater and interrater agreement

There was an excellent intra-rater agreement for both baseline (ICC 0.976, *p* < 0.001) and follow-up imaging (ICC 0.965, *p* < 0.001). In line, the interrater agreement was excellent for baseline (ICC 0.943, *p* < 0.001) and follow-up imaging (ICC 0.971, *p* < 0.001).

## Discussion

Our study showed four major findings. First, temporal changes of the ASPECTS were – as expected – more favorable in patients with successful recanalization. Second, successful recanalization had a clear effect on the cortex but not on the deep brain structures, which were more prone to new ischemic changes at 24 h. Third, the effect of recanalization was seen in the ICA/M1 subcohort, but not in the M2 occlusions. Finally, we have not observed any region in any patient, in which early ischemic change would revert after complete recanalization.

Early ischemic changes NCCT are related to pathophysiology of cerebral edema, involving water homeostasis, inflammatory changes, cell membrane transport, and blood brain barrier (BBB) disruption.^12^ Correctly assessing true infarct core can be challenging.^13^ There are three described phases of edema evolution in stroke pathophysiology. Early cytotoxic edema occurs minutes after cessation of blood flow and via cellular membrane dysfunction leads to cell swelling and is detectable by MRI DWI.^14^ In this phase, cellular membrane dysfunction causes swelling of the cells (mainly neuronal dendrites and astrocytes) due to water shifts from extracellular to intracellular leading to signal changes on DWI. This process per se does not represent overall net water content in tissue, but it causes ionic gradient between interstitial fluid space and vascular compartment. This gradient leads to the second phase, so-called ionic edema, where BBB is still intact but net water content of tissue starts to rise. Water uptake in ischemic tissue is still maintained from residual or collateral blood flow and from cerebrospinal fluid. Once BBB is disrupted (typically after 4–6 h), blood products (first mainly water and plasma proteins) start to leak into the tissue increasing its water content resulting in vasogenic edema. Ionic edema is the main reason for tissue net water uptake and thus hypoattenuation on NCCT detected in the acute phase leading to an approximately decrease of 2–4 Hounsfield Unit in first 3 h onset from stroke.^15^ Also, severity of hypoattenuation in infarcted tissue is correlated with worse prognosis,^16^ and malignant edema.^17^

In our study, no reversal of hypoattenuation was observed and all baseline ischemic changes were also detectable at 24 h (and also in any subsequent CT scan taken during the hospitalization). The same phenomenon was described in the early 2000s.^5^ Since then, we have witnessed a massive evolution of CT technology. Firstly, late 90s multidetector CTs were introduced to a broader clinical use, wide-cone CT came later, and spectral CT (dual energy) is widely used nowadays. The quality of CT detectors has also improved with spatial resolution being currently 0.3 mm or even less. Image acquisition algorithms have noticeably improved over the last 20 years. From 2009 iterative reconstruction algorithm was introduced with help of modern computers and high computational power. With modern evolved detectors and image reconstruction algorithms, we can achieve better signal- and contrast-to-noise ratio with acceptable radiation dose compared to groundwork studies performed late 90s and early 2000. The currently common availability of different reconstruction planes also facilitates detection subtle signs of early ischemia.^7,8^

Our study was conducted with technologically more advanced scanners than those from the late 90s and early 2000s. Yet, we have not noticed any reversal of ischemic changes in any brain region. Indeed, our results are in line with previous research performed on CT scanners >20 years ago.^5^ Minimal reversal of DWI changes [median absolute reversible volume 1 ml (0–2)] was very recently reported by Scheldeman et al.,^18^ whereas Albach et al.^19^ showed that reversal of DWI hyperintensities after ischemic stroke is limited to small embolic lesions. Based on experimental data, it has been shown that cytotoxic edema (DWI) occurs at cerebral blood flow levels that are above levels, which cause permanent cell death. In contrast, ionic edema (NCCT hypoattenuation) is driven by such cerebral blood flow levels that rapidly lead to irreversible neuronal dysfunction and cell death.^20^ Hence, this difference in initial blood flow levels between cytotoxic an ionic phase might be one of the reasons why no reversal was observed in NCCT despite complete recanalization in our cohort. Although successful recanalization was previously linked to ischemia-related edema evolution,^21^ it was observed in the context of vasogenic edema, which is a secondary edema after ischemia but does not represent an irreversible change.

EXTEND-IA^22^ reported median times of 93 (IQR 71–138) minutes from imaging to groin puncture and 43 (24–52) minutes from puncture to mTICI 2B/3. DEFUSE-3^23^ reported median times of 59 (IQR 39–87) minutes from imaging to puncture and 38 (IQR 26–59) minutes to recanalization. Our delays from initial Imaging to recanalization mTICI 2B/3 are shorter than EXTEND-IA and comparable to DEFUSE-3. In our study, even those with shortest delays in recanalization or FPE showed no EIC reversibility.

Poor recanalization (mTICI 0–1 and 2A) in our cohort led – as expected – to significant temporal decline of total ASPECTS compared to complete recanalization (mTICI 3), however, there was only a strong trend in comparison between mTICI 2B and mTICI 3 (Table 2, all patients). It is possible that if we had further dissected mTICI 2B into 2B and 2C, we would have seen significant difference for 2B but not for 2C. Furthermore, in each of the ASPECTS subregions, there was a significant increase of ischemic burden between baseline and 24 h irrespective of recanalization (Table 3, left). Including recanalization status in the analysis, only the cortex showed a “protective” (but not reversible) effect of recanalization (Table 3, right). For the insular region, we observed only a trend toward more frequent ischemia in mTICI 0–1 subcohort (compared with mTICI 3) but mTICI 2A and 2B were not different from mTICI 3. In lentiform and caudate nuclei as well as capsular regions, development of ischemic changes between baseline and 24 h was similar among all recanalization patterns.

The observation that basal ganglia and insular cortex are most prone to ischemia was observed also by others.^24^ This vulnerability can at least partially be explained by factors contributing to the lack of proper collateral flow. For instance, the insular cortex is primarily supplied by M2 segments, distant from potential ACA or PCA leptomeningeal anastomosis. The basal ganglia are mainly supplied from M1 through perforators (lenticulostriate arteries), which lack sufficient collaterals. It is understandable that more distal occlusions would typically lead to infarcts that are smaller than those caused by large vessel occlusion. That can partly explain why temporal change of ASPECTS was influenced by recanalization in M1/ICA but not in M2 segments in our cohort. Furthermore, the number of M2 occlusions in our cohort was considerably smaller compared to ICA/M1.

In general, we observed a significant change toward more common presence of ischemic changes at 24 h compared with baseline, both in total ASPECTS and in its subregions (Tables 1 and 3, Figure 1). From Figure 1, we can see that there were some patients (*n* = 36) with high ASPECTS (8–10) at 24 h despite poor recanalization (Figure 1). One patient had an ASPECTS of 10 on initial NCCT but 5 on day 7 discharge imaging (without indications of a new ischemic event). Another patient had minor cortical infarcts only detected by MRI on day 5. Fourteen of them had ipsilateral ICA or M1 stenosis over 50%, which probably translated into better collateral flow, however, majority (*n* = 20) had distal M2 thrombus at baseline

It is well known that the mTICI classification is simpler to use in proximal anterior circulation stroke than in distal locations.^25^ To overcome some of the challenges with mTICI classification, our reference was vessel occlusion detected on DSA at the start of EVT.^26^ This is very important, because thrombus in M1 (on baseline CTA) can migrate distally to M2 segment before EVT.^27^ In such a case, it would be misleading to state that mTICI is 2B. In this situation even after successful (but not complete) recanalization, mTICI would not necessarily change although the reperfusion status is obviously better than on the initial CTA and at the beginning of EVT as seen on DSA. That is why some reports classify mTICI as related to the whole MCA perfusion status, and not to the occluded vessel per se (unless it is M1). Typical example would be a persistent occlusion in M3 branch, which can be correctly classified as mTICI 0 (per occluded vessel approach) or as 2B (per the whole MCA perfusion status). Of note, the presence of ischemia in all ASPECTS territories in some of the M2 patients can also be explained by migration of M1 thrombus to more distal location.

One limitation of our study can be the visual assessment of ischemic changes, which is to some extent subjective and has some degree of variability.^28^ ASPECTS scoring can lead to overestimation of infarct severity.^29^ Defining exact infarcted parenchyma can be challenging with both visual and quantitative based on automatic software due to the inevitable noise and very subtle EIC in CT images.

Post EVT parenchymal enhancing also affects NCCT evaluation.^30^ This is problematic both for visual and quantitative methods such as measurement of net water uptake.^31^ To minimize variability, in our study, all the scans were evaluated by the same neuroradiologist with an excellent intra- and inter-rater rater correlation. Further, we had a subcohort of nearly 100 patients with 24-h dual-energy CT imaging. This allowed for better detection of ischemia after treatment and distinguished bleeding from contrast enhancement.^32^ Finally, in addition to systematic evaluation of the 24-h scans, we have also evaluated all other available scans acquired during the whole length of hospitalization, which gave us a more refined picture of the final infarct size. Another limitation might be the slight variability in scanner models between initial and subsequent imaging sessions. However, all follow-up CT scans were performed using Siemens (Force or EDGE) scanners, employing same imaging algorithms.

## Conclusions

Temporal evolution of the ASPECTS is dependent on the success of the recanalization after EVT. Effect of recanalization on these temporal changes was observed only in the cortical regions of patients with ICA/M1 occlusions, but not in their deep brain structures or in any region of M2 occlusion patients. Finally, we did not observe any brain region in any of the patients, in whom early ischemic change would revert after successful recanalization.
